# Constructing Bi_24_O_31_Cl_10_/BiOCl heterojunction via a simple thermal annealing route for achieving enhanced photocatalytic activity and selectivity

**DOI:** 10.1038/srep28689

**Published:** 2016-06-24

**Authors:** Xiaoyan Liu, Yiguo Su, Qihang Zhao, Chunfang Du, Zhiliang Liu

**Affiliations:** 1 College of Chemistry and Chemical Engineering, Inner Mongolia University, Hohhot, Inner Mongolia 010021, P. R. China

## Abstract

This work reports on the construction of a Bi_24_O_31_Cl_10_/BiOCl heterojunction via a simple thermal annealing method. The X-ray diffraction (XRD) results indicated that the phase transformation from BiOCl to Bi_24_O_31_Cl_10_ could be realized during the thermal annealing process. The high-resolution transmission electron microscopy (HRTEM) images, X-ray photoelectron spectroscopy (XPS) binding energy shifts, Raman spectra and Fouier transform infrared spectroscopy (FT-IR) spectra confirmed the formation of the Bi_24_O_31_Cl_10_/BiOCl heterojunction. The obtained Bi_24_O_31_Cl_10_/BiOCl photocatalyst showed excellent conversion efficiency and selectivity toward photocatalytic conversion of benzyl alcohol to benzaldehyde under visible light irradiation. The radical scavengers and electron spin resonance (ESR) results suggested that the photogenerated holes were the dominant reactive species responsible for the photocatalytic oxidation of benzyl alcohol and superoxide radicals were not involved in the photocatalytic process. The *in-situ* generation of Bi_24_O_31_Cl_10_/BiOCl heterojunction may own superior interfacial contact than the two-step synthesized heterojunctions, which promotes the transfer of photogenerated charge carriers and is favorable for excellent photocatalytic activities.

Regarding the future environmental and energy concerns, the development of green and sustainable chemical conversions has attracted enormous interest^1^,^2^,^3^. Alcohol oxidations are one of the most frequently investigated reactions because of their industrial essentiality in the commercial synthesis of multifarious materials, such as plastics, perfumes, paints, etc^4^,^5^,^6^. Compared with conventional methods, photocatalytic technology is considered to be a green, reliable and economic method for the oxidation of alcohols into the corresponding aldehydes due to the massive solar energy and O_2_^7^,^8^,^9^,^10^,^11^.

Semiconductor titanium dioxide (TiO_2_) is universally regarded as an efficient photocatalyst toward decomposition of various organic pollutants^12^,^13^,^14^,^15^,^16^,^17^. Moreover, it also displays photocatalytic activity toward the oxidation of benzyl alcohol to benzaldehyde under UV-light and visible-light irradiation, which shows high conversion efficiency (>99%) and selectivity (>99%)^18^,^19^. Recently, considerable attention has been devoted to another series of semiconductors, the bismuth-based semiconductors. BiOCl is a V-VI-VII ternary semiconductor, consisting of internal structure of [Bi_2_O_2_]^2+^ layers sandwiched by two slabs of Cl atoms which induces the growth of BiOCl along a particular axis^20^. It often shows high photocatalytic performance than TiO_2_ (P25, Degussa) under UV-light irradiation due to its unique layered atomic structure, which favors the transfer and separation of photogenerated charge carriers and subsequently enhances the photocatalytic activity^21^,^22^. However, BiOCl is a wide-band-gap (3.17 ~ 3.54 eV) semiconductor^23^,^24^, which leads to a poor photocatalytic performance under visible light irradiation.

Constructing heterojunction composed of BiOCl and another narrow band-gap semiconductor with suitable conduction band (CB) and valence band (VB) can efficiently improve the visible-light harvesting and inhibit the electron-hole recombination as well as raise the lifetime of charge carriers. A variety of heterojunction systems containing BiOCl and a narrow band-gap semiconductor has been intensively investigated, e.g. g-C_3_N_4_/BiOCl^25^, Bi_2_S_3_/BiOCl^26^, BiOI/BiOCl^27^, CdS/BiOCl^28^, WO_3_/BiOCl^29^, BiVO_4_/BiOCl^30^, NaBiO_3_/BiOCl^31^, etc. All these heterojunctions presented enhanced photocatalytic performances than their single-component counterparts.

From the viewpoint of solid state physics, details of the band edge potential are primarily determined by the static potential within the unit cell of a semiconductor^32^. Any symmetry and component perturbations can have consequence on the electronic structures and physical properties. Since the potential of conduction band minimum (CBM) and valence band maximum (VBM) are mainly related to Bi 6p and Bi 6s orbitals respectively, the regulation of CBM and VBM of bismuth-based semiconductors can be achieved by adjusting the Bi content^33^,^34^. Recently, nontypical stoichiometric semiconductors (NSSs), including Bi_3_O_4_Cl^35^, Bi_12_O_15_Cl_6_^36^, Bi_24_O_31_Cl_10_^33^ have been found to show visible light driven photocatalytic activities, which are regarded as ideal candidates for the construction of heterojunctions with BiOCl. These NSSs have narrower band gap, faster transfer of charge carriers and more efficient separation of photogenerated electron-hole pairs^37^. Furthermore, they have the approximate crystalline architecture relative to their corresponding typical stoichiometric semiconductors (TSSs). As a nontypical stoichiometric bismuth-based semiconductor, Bi_24_O_31_Cl_10_ is widely known as a product of the thermal decomposition of BiOCl^38^. It has a narrow band gap of about 2.7 ~ 2.8 eV^33^,^39^, demonstrating a good visible-light harvesting. Thus, Bi_24_O_31_Cl_10_/BiOCl heterojunction may be a promising photocatalyst in the visible light region, if both of them have the suitable CB and VB levels^40^.

In the present study, Bi_24_O_31_Cl_10_/BiOCl heterojunction was constructed via a *in-situ* fabrication. Although Bi_24_O_31_Cl_10_ is widely known as a thermal decomposition product of BiOCl, the structure and the photocatalytic performance of the intermediate product Bi_24_O_31_Cl_10_/BiOCl heterojunction were not investigated in detail. The oxidation of benzyl alcohol to benzaldehyde is firstly chosen as the model reaction to check the photocatalytic performance of the Bi_24_O_31_Cl_10_/BiOCl heterojunction. The *in-situ* fabrication of Bi_24_O_31_Cl_10_/BiOCl heterojunction may predict more interfacial contact for efficient charge carriers separation^37^, resulting in highly enhanced photocatalytic performance toward benzyl alcohol oxidation.

## Results and Discussion

**Figure 1:**
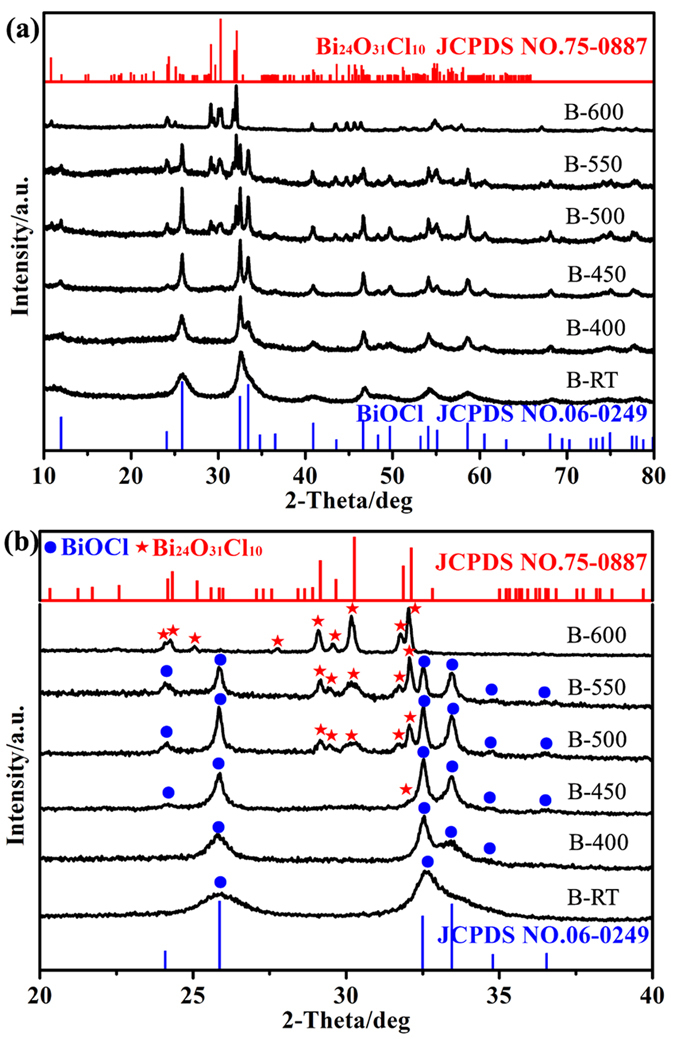
Wide and enlarged XRD patterns of various samples.

Figure 1 displays the XRD patterns of BiOCl and the calcined samples. The XRD pattern of sample B-RT is assigned to tetragonal BiOCl (JCPDS NO. 06-0249). With an increase of annealing temperature, the XRD peaks belonging to Bi_24_O_31_Cl_10_ with a monoclinic structure (JCPDS NO. 75-0887) emerges. No apparent diffraction peaks belonging to BiOCl are observed when the temperature increased up to 600 °C. The enlarged XRD patterns of all samples in the range of 2*θ* = 20 ~ 40° are also presented to further verify the transformation process from tetragonal BiOCl to monoclinic Bi_24_O_31_Cl_10_ (Fig. 1b). A weak peak located at 32° is assigned to Bi_24_O_31_Cl_10_ in XRD pattern of sample B-450. The other three typical strong peaks nearby 30° are observed in sample B-500, which indicates that large amount of Bi_24_O_31_Cl_10_ is produced at reaction temperature of 500 °C. Further increase of reaction temperature induces the emergence of more diffraction peaks belonging to Bi_24_O_31_Cl_10_ and all the XRD peaks belonging to Bi_24_O_31_Cl_10_ phase are only left at 600 °C (B-600). On the other hand, no XRD peak of Bi_24_O_31_Cl_10_ phase is observed in sample B-400, which may suggest that no observable phase transformation occurs or the Bi_24_O_31_Cl_10_ does not possess sufficient long-range order to be checked by XRD. DTA-TG curves (Figure S1) of sample B-RT shows that there is an exothermic peak at about 400 °C, suggesting that the phase transformation of BiOCl occurs as the temperature achieving to 400 °C, which result is consistent with the XRD results.

**Figure 2:**
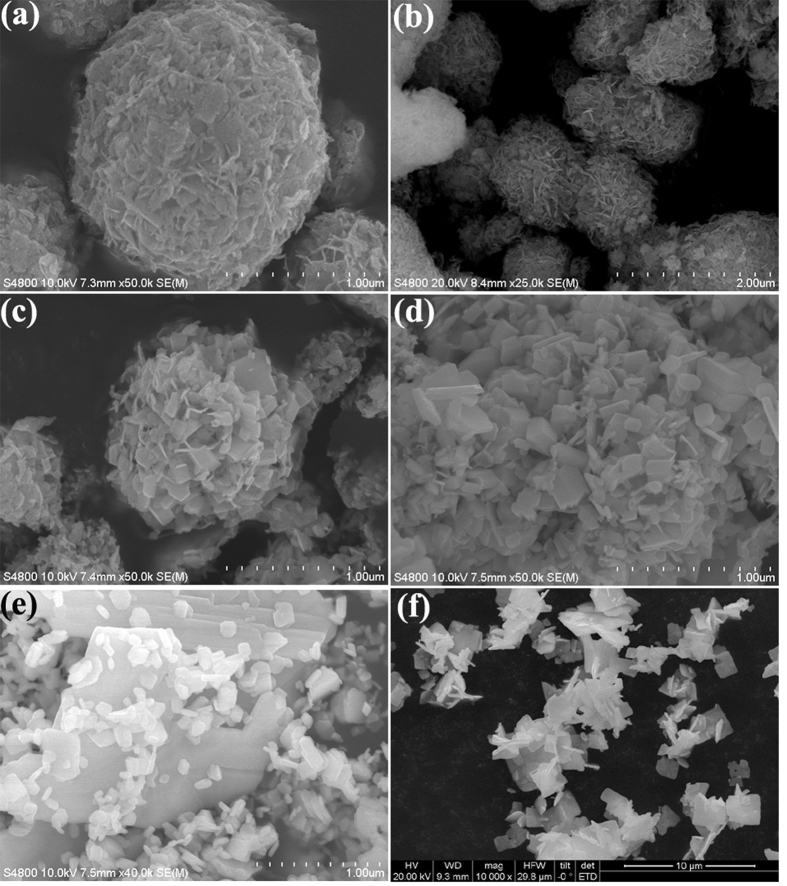
SEM images of B-RT (**a**), B-400 (**b**), B-450 (**c**), B-500 (**d**), B-550 (**e**), B-600 (**f**).

Figures 2 and S2 shows the SEM images of various samples. The image in Fig. 2a displays that the pure BiOCl spheres with diameter of about 1.5 ~ 2.0 μm are mainly consisted of irregular nanosheets, which are 0.1 ~ 0.2 μm in width and 3 ~ 5 nm in thickness (Figure S2a). After calcination at 400 °C, the nanosheet edges and angles of sample B-400 are distinct and differentiable (Fig. 2b). The gradual increased temperature leads to the morphological transformation from compact sphere to loose structure as well as irregular nanosheets to square analogs (Fig. 2c,d). Furthermore, the nanosheets of BiOCl become wider and thicker with an increase of annealing temperature. Sample B-600 (pure Bi_24_O_31_Cl_10_) presents square-like plate structure with 1 ~ 2μm in width and ~0.1 μm in thickness (Figs 2f and S2b). It could also be observed that the sheet-shaped structure with narrower width and thinner thickness decreases, while the plate-shaped structure increases by elevating the annealing temperature, which result is consistent with the BET results (Figure S3) that sample B-600 has the lower specific surface area (*S*_BET_) than sample B-RT.

**Figure 3:**
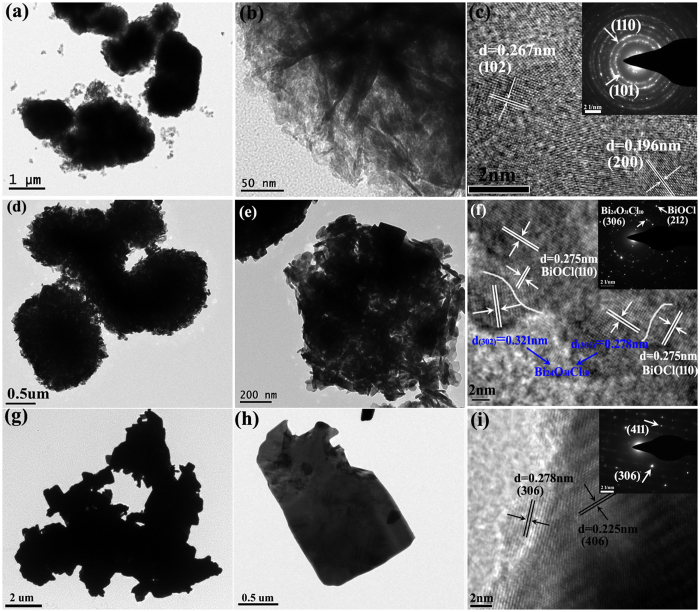
TEM, HRTEM images and SAED patterns of samples B-RT(**a**–**c**), B-450 (**d**–**f**) and B-600 (**g**–**i**).

The detailed morphologies, crystal structures and the heterojunction features of samples B-RT, B-450 and B-600 are characterized by TEM, HRTEM and SAED. Figure 3a,b reveal that BiOCl spheres are composed of irregular nanosheets, which is consistent with the SEM observation (Fig. 2a). HRTEM image in Fig. 3c discloses that the distances between the adjacent lattice fringes are about 0.267 and 0.196 nm, matching well with the (102) and (200) crystalline plane of BiOCl, respectively. The selected area electron diffraction (SAED) (Inset of Fig. 3c) clearly presents the crystalline planes of (101) and (110) of BiOCl, respectively. Sample B-450 keeps the same diameter, but the shape of the nanosheets becomes regular (Fig. 3d,e). Figure 3f provides a comprehensive information of the Bi_24_O_31_Cl_10_/BiOCl heterojunction. The lattice fringes with the *d* spacing of 0.275 nm correspond to the (110) crystalline plane of BiOCl, whereas the lattice fringes with the *d* spacing of 0.321 and 0.278 nm belong to the (30-2) and (306) crystalline plane of Bi_24_O_31_Cl_10_, respectively. Furthermore, as displayed in Fig. 3f that there exists an identifiable interface (presented by white line) and continuity of the lattice fringes between BiOCl and Bi_24_O_31_Cl_10_, indicating the formation of a heterojunction between the two semiconductors. The SAED pattern (Inset of Fig. 3f) further confirms the coexistence of BiOCl and Bi_24_O_31_Cl_10_. Figure 3g,h reveal that sample B-600 displays a square-like structure and no apparent BiOCl spheres are observed. Both the HRTEM image and the SAED pattern in Fig. 3i indicate the single-crystalline characteristic of Bi_24_O_31_Cl_10_.

**Figure 4:**
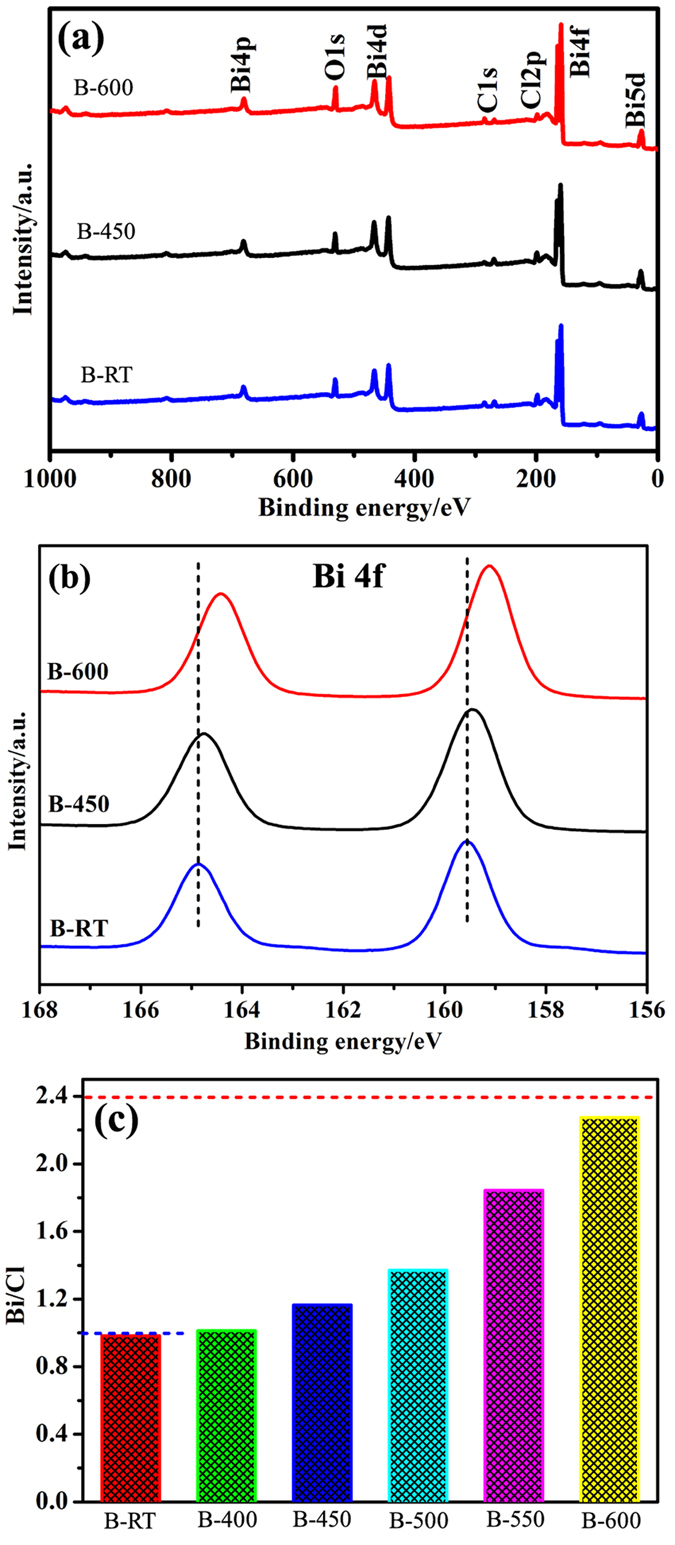
XPS survey spectra (**a**) and high-resolution XPS spectra for Bi 4f (**b**) of samples B-RT, B-450 and B-600, and the variation of Bi/Cl molar ratio as a function of reaction temperature (**c**). The blue and red dash lines are the theoretical values of Bi/Cl molar ratio for pure BiOCl and Bi_24_O_31_Cl_10_, respectively.

To further confirm the chemical state and chemical composition of the as-prepared samples, X-ray photoelectron spectroscopy (XPS) analysis was applied and the results are shown in Fig. 4. The survey scans of samples B-RT, B-450 and B-600 distinctly reveal the co-existence of Bi, O and Cl elements without other impurities, excluding adventitious carbon-based contaminant. The two primary peaks at ~159.0 eV and ~164.0 eV in Bi 4f XPS spectra result from the spin orbital splitting photoelectrons of Bi 4f_7/2_ and Bi 4f_5/2_, respectively^41^. There is an obvious red-shift in the Bi 4f binding energy with increasing the temperature to 600 °C. Variations in the elemental binding energies are generally related to the difference in chemical potential and polarizability of involved elements^42^,^43^. Thus, the binding energy shift in sample B-450 is possibly attributed to the interaction between BiOCl and Bi_24_O_31_Cl_10_, which result is similar to the SnO_2−x_/g-C_3_N_4_^44^ and TiO_2_/ZnPcGly^45^. It is reported that the increase or decrease in electron concentration could enhance or reduce the electron screening effect, which would weaken or strengthen the binding energy^46^. The higher electronegativity of Bi could induce increased electron concentration in the new formed bonds^37^, such as Bi-Cl or/and Bi-O bands at the interface, which enhances the electron screening effect and leads to the Bi 4f peaks shift toward lower binding energy. Furthermore, the position of Bi 4f peaks in sample B-600 is also different from that in sample B-RT, which could be attributed to the different chemical environment of Bi ions in BiOCl and Bi_24_O_31_Cl_10_. This observation is in accordance with the XPS results of BiOCl/Bi_12_O_15_C_l6_^36^ and BiVO_4_/Bi_4_V_2_O_11_^37^. However, the span between the two binding energy peaks maintains the same value of 5.3 eV, which suggests that Bi exists in the chemical state of Bi^3+^ in both BiOCl and Bi_24_O_31_Cl_10_.

The chemical compositions of Bi, Cl and O in various samples as well as the variation of Bi/Cl molar ratio as a function of annealing temperature are displayed in Fig. 4c and Table S1. As shown in Fig. 4c, there exists a monotonic increase of Bi/Cl molar ratio with an increase of annealing temperature. When the temperature increases to 600 °C, the Bi/Cl molar ratio reaches 2.295, which is very close to the theoretical value 2.4 of Bi_24_O_31_Cl_10_. This observation indicates the phase transformation from pure BiOCl to Bi_24_O_31_ Cl_10_. It could be possibly accepted that if the Bi/Cl molar ratio is larger than the theoretical value of BiOCl, the phase transformation occurs. Thus, 450 °C could be recognized as the initial phase transformation temperature in our experiment, which is consistent with the XRD result.

**Figure 5:**
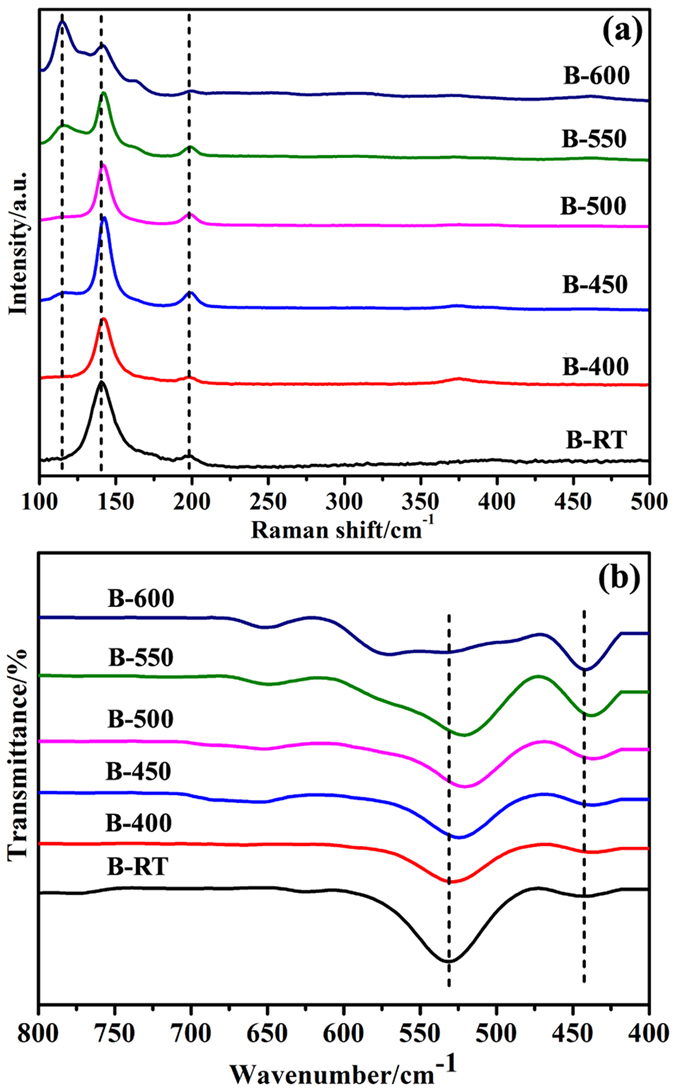
Raman spectra (**a**) and FT-IR spectra (**b**) of various samples.

Raman and FT-IR measurements are performed to investigate the BiOCl phase transformation and interfacial interactions between BiOCl and Bi_24_O_31_Cl_10_. For sample B-RT (Fig. 5a), there are two distinguishable Raman active bands at 140 cm^−1^ and 198 cm^−1^ which are assigned to the A_1g_ and E_g_ internal Bi-Cl stretching modes^47^,^48^, respectively. However, the band related to the motion of oxygen atoms at about 400 cm^−1^ 36,49 is very weak and nearly unnoticeable. With an increase of annealing temperature, the Raman peak assigned to A_1g_ shifts to higher wavenumbers. This phenomenon could be ascribed to the formation of heterojunction between BiOCl and Bi_24_O_31_Cl_10_, because the interfacial contact might produce intrinsic stresses on the crystal structure and alter the periodicity of the lattice^37^,^50^. However, for sample B-600, there exists a new band located at 115 cm^−1^, which is close to that of pure Bi_24_O_31_Cl_10_ (Figure S4)^33^, suggesting the presence of Bi_24_O_31_Cl_10_ in sample B-600.

Figures 5b and S5 show the FT-IR spectra of samples B-RT ~ B-600. For sample B-RT, the peaks at 3437 cm^−1^ and 1622 cm^−1^ in Figure S5 are assigned to the stretching vibration and deformation vibration of the hydroxyl group (−OH) acquired from the wet atmosphere^51^. The band at 2925 cm^−1^ represents the C-H stretching vibration^52^, which originates from glycerol in the synthetic procedure of BiOCl. The bands striding over the wavenumbers 1036 to 1406 cm^−1^ are ascribed to the stretching vibration of the C-O-C bond in glycerol^52^. The bands located between 200 ~ 800 cm^−1^ correspond to the characteristic of Bi-O bond, and the peak at about 523 cm^−1^ resulted from the symmetrical stretching vibration of the Bi-O band is a typical peak of BiOCl^51^,^53^,^54^. With an increase of the annealing temperature, the weakening of the bands assigned to −OH, C-H and C-O-C is attributed to the gradual removal of adsorbed water and glycerol (Figure S5). Furthermore, it can be identified in Fig. 5b that the band at 523 cm^−1^ exhibits a blue shift and the peak located at 442 cm^−1^ is gradually distinguishable, verifying the interfacial interactions caused by the construction of the heterojunction between BiOCl and Bi_24_O_31_Cl_10_ as well as the dominant existence of Bi_24_O_31_Cl_10_, which result is similar to that of BiVO_4_/Bi_4_V_2_O_11_^37^. Based on the results from HRTEM, XPS, Raman and FT-IR spectra, it could be concluded that the Bi_24_O_31_Cl_10_/BiOCl heterojunction is successfully constructed, which is probably helpful for the transfer and separation of photogenerated charge carriers as well as the improvement of photocatalytic activity.

**Figure 6:**
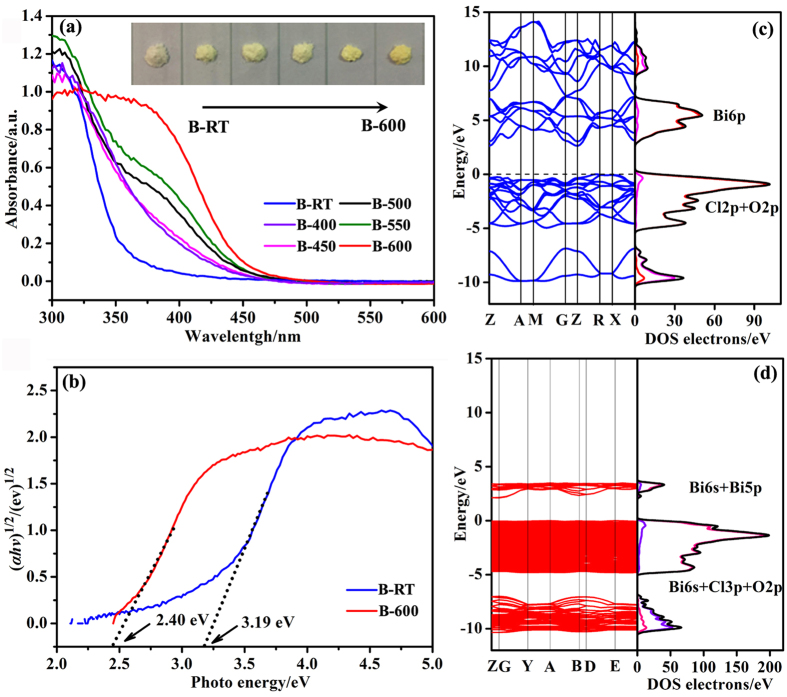
UV-vis diffuse reflectance spectra (DRS) (**a**) and plots of (*αhv*)^1/2^ vs. the photo energy (*hv*) (**b**) for samples, calculated band structure and density of states (DOS) of BiOCl (**c**) and Bi_24_O_31_Cl_10_ (**d**).

The photocatalytic performance of catalysts is related to the light absorption, thus the UV-vis diffuse reflectance spectroscopy (DRS) was adopted to determine the visible light harvesting ability of BiOCl and calcined samples (Fig. 6a). BiOCl presents almost no absorption in the visible light region with an absorption edge at 360 nm. Interestingly, there exists a red shift of the absorption edge with an increase of the annealing temperature, and the sample B-600 (pure Bi_24_O_31_Cl_10_) possesses the most intense visible light harvesting ability with an absorption edge at about 455 nm. It should be noted that samples B-500 and B-550 exhibit similar absorption feature in comparison with pure Bi_24_O_31_Cl_10_ (B-600), this result is in accordance with the XRD result that massive Bi_24_O_31_Cl_10_ phase exists in sample B-500. The new emerged absorption edge also indicates that Bi_24_O_31_Cl_10_/BiOCl heterojunction photocatalyst should display visible light photocatalytic activity. The UV-vis spectra result is also confirmed by the colors of BiOCl (B-RT) and calcined samples (B-400 ~B-600), changing from white to yellow, as shown in inserted graph in Fig. 6a.

It is accepted that the band gap energy of a semiconductor can be evaluated by the following equation:


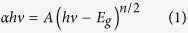


where *α, v, E*_g_, and *A* are the absorption coefficient, light frequency, band gap energy, and a constant, respectively. The parameter *n* is determined by the characteristics of the transition in a semiconductor (i.e., n = 1 for direct transition or *n* = 4 for indirect transition). In order to specify the *n* values of BiOCl and Bi_24_O_31_Cl_10_, the density functional theory (DFT) calculations are carried out (Fig. 6c,d). The calculated Fermi level is set at an energy of zero eV in the band gap, indicating typical intrinsic semiconducting characteristics in the electronic structure. Fig. 6c (left) shows that the conduction band minimum (CBM) and the valence band maximum (VBM) are located at Z and R point, respectively. It indicates that BiOCl is an indirect band gap semiconductor with a band gap of 2.63 eV, which is close to the previous DFT calculations^55^,^56^. The calculated band structure and density of states (DOS) (Fig. 6c right) imply that the CB of BiOCl mainly consists of Bi6p orbitals, whereas the VB is contributed by hybridized Cl2p and O2p orbitals. It could be inferred from Fig. 6d that Bi_24_O_31_Cl_10_ is also an indirect band gap semiconductor with a band gap of 2.11 eV, which is consistent with the previous DFT calculations^39^. The CB of Bi_24_O_31_Cl_10_ mainly consists of Bi6s and Bi5p orbitals, whereas the VB has major contribution from the hybridized Bi6s, Cl3p and O2p orbitals.

Having these results in mind, the *n* values for both BiOCl and Bi_24_O_31_Cl_10_ are 4. Thus, the band gap energies of pure BiOCl and Bi_24_O_31_Cl_10_ could be estimated from a plot of (*αhv*)^1/2^ versus the photon energy (*hv*). The intercept of the tangent to the x-axis will give a good approximation of the band gap energies for various samples. As shown in Fig. 6b, the optical band gaps of sample B-RT and B-600 are calculated to be 3.19 eV and 2.40 eV, respectively, which are close to the previously reported values^33^,^40^,^57^.

**Figure 7:**
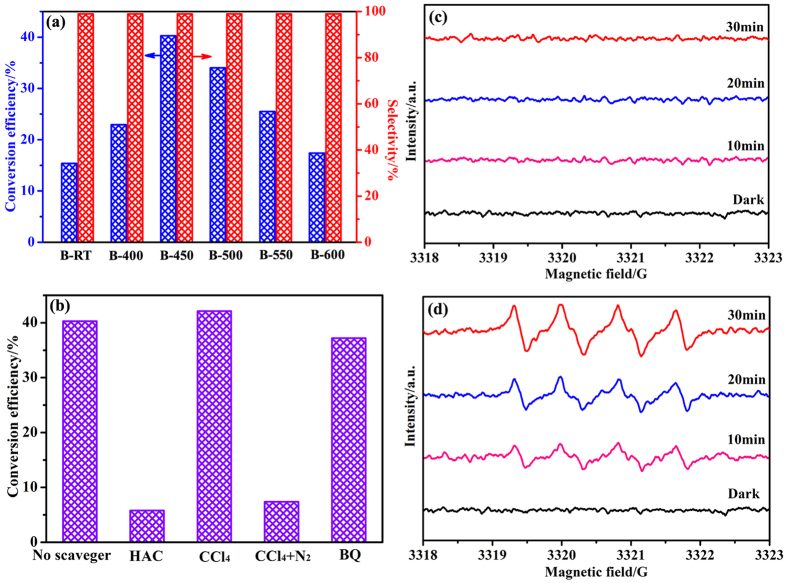
Photocatalytic conversion of benzyl alcohol over various samples under visible light irradiation (**a**), effects of scavengers on the conversion of benzyl alcohol (**b**), DMPO spin-trapping ESR spectra of sample B-450 in methanol dispersion for DMPO-^·^OH (**c**) and in 20% methanol +80% methylbenzene dispersion for DMPO-^·^O_2_^−^ (**d**).

It is accepted that the selective photocatalytic oxidation of benzyl alcohol to benzaldehyde using O_2_ as the oxidizing agent is considered as a model reaction to evaluate the photocatalytic performance of semiconductors^58^. Figure 7a displays the benzyl alcohol conversion efficiency over various samples. Notably, all samples exhibit photocatalytic activities toward benzyl alcohol oxidation under visible light irradiation. It’s noted that pure BiOCl (B-RT) with a band gap of 3.19 eV also shows a benzyl alcohol conversion efficiency of 15.4%. TiO_2_, as a wide band-gap semiconductor, also displays excellent conversion efficiency (>99%) and selectivity (>99%) toward benzyl alcohol oxidation under visible light irradiation. This phenomenon is ascribed to the corresponding absorption edge shifts and absorption intensity enhancement in the visible-light region, which is related to the formation of a visible-light responsive charge-transfer complex between TiO_2_ and benzyl alcohol^18^,^19^. To specify the reason that BiOCl exhibits visible light photocatalytic activity toward benzyl alcohol oxidation, UV-vis absorption spectra of benzyl alcohol (BA)-adsorbed samples are investigated (Figure S6). As illustrated in Figure S6, there is nearly no obvious changes in absorption edges and intensities in visible-light region for both BA-adsorbed samples and bare samples. Thus, it is expected that the benzyl alcohol conversion efficiency over the present samples may be not related to the charge-transfer complex formed between photocatalysts and benzyl alcohol. The photocatalytic activity of BiOCl under visible light irradiation may be related to the special nanosheet structure and 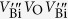 vacancy associates in BiOCl^23^,^59^. The conversion efficiency reaches a maximum of approximately 40.3% with increasing the annealing temperature to 450 °C, however, further increase of the annealing temperature leads to an obvious decrease in the conversion efficiency. Furthermore, all samples display >99% selectivity toward benzaldehyde. Although the photocatalytic performance of the as-prepared Bi_24_O_31_Cl_10_/BiOCl heterojunction is lower than that of TiO_2_ and Na_*x*_TaO_*y*_·*n*H_2_O^1^,^19^, it is close to even higher than several oxyhalides, such as Bi_3_O_4_Br, BiOBr and Bi_12_O_17_Cl_2_^7^ (Table S2), suggesting comprehensive work needs to be further conducted for oxyhalide semiconductors in the future.

It is well known that the photocatalytic process involves the photogenerated electrons and holes, which could react with the molecular O_2_ and H_2_O/HO^−^ to yield superoxide radical (^·^O_2_^−^) and ^·^OH, respectively. The new produced active species are essentially important in the catalytic reactions. To reveal the origin of the highly photocatalytic performance and selectivity for the Bi_24_O_31_Cl_10_/BiOCl heterojunction, a series of active species trapping experiments were further conducted and the results are displayed in Fig. 7b. When acetic acid (HAC) as holes scavenger is added, the conversion efficiency of benzyl alcohol decreases significantly. The addition of tetrachloromethane (CCl_4_) and benzoquinone (BQ) used as an electron and superoxide radical scavenger respectively, makes a slight influence in the conversion efficiency. These observations suggest that photogenerated holes act as the dominant role in the photocatalytic conversion of benzyl alcohol. Moreover, if molecular nitrogen is used instead of molecular O_2_ in the presence of CCl_4_ during the photocatalytic process, the conversion efficiency surprisingly decreases, which suggests that molecular O_2_ is specially vital in the photocatalytic reaction. That is to say, the generation of superoxide radicals, which consumes lots of the photogenerated electrons, could greatly inhibit the recombination of photogenerated charge carriers, favoring the selective oxidation of benzyl alcohol to benzaldehyde originated by photogenerated holes.

The above result could also be proved by ESR technique. DMPO spin-trapping ESR spectra of sample B-450 to reveal the generation of active species ^·^O_2_^−^ and ^·^OH are displayed in Fig. 7c and d. As shown in Fig. 7c, no characteristic ESR signal is detected either in the dark or in the visible light irradiation from 10 min to 30 min, indicating that ^·^OH is not involved in the photocatalytic process. In Fig. 7d, there is no characteristic ESR signal observed in dark. However, the characteristic peaks of DMPO-^·^O_2_^−^ adduct are detected after 10 min of visible light irradiation. Furthermore, the intensity of the DMPO-^·^O_2_^−^ signals increases with prolonging the irradiation time.

Combining the results of scavengers experiment and ESR spectra, it could be concluded that the photogenerated holes are the major active species in the photocatalytic conversion of benzyl alcohol under visible light irradiation, the active species ^·^O_2_^−^ are indeed formed during the photocatalytic process but not involved in the photocatalytic reaction. For potential applications, the stability of the heterojunction photocatalyst should be taken into consideration. Figure S7 presents the XRD patterns of sample B-450 before and after photocatalytic process. There is no structural variation between the samples before and after catalytic reaction, indicating the strong structural stability of Bi_24_O_31_Cl_10_/BiOCl heterojunction.

**Figure 8:**
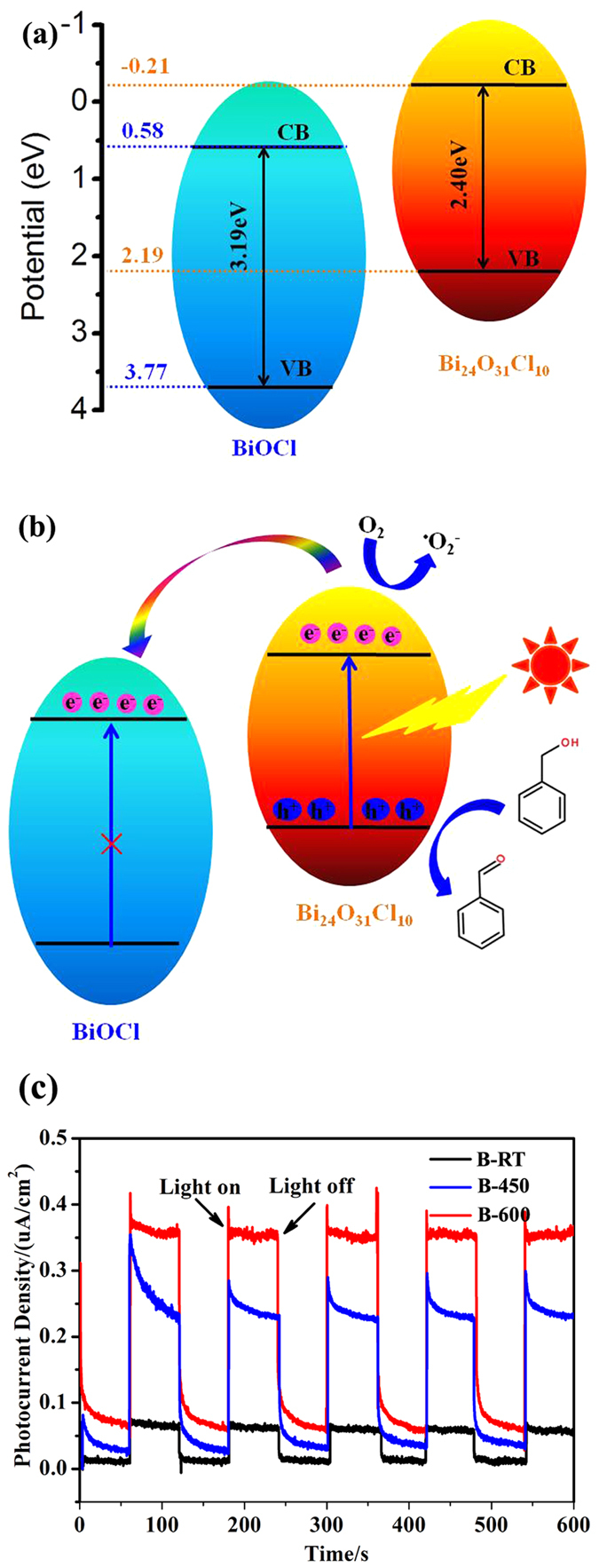
The schematic band diagrams of pure BiOCl and Bi_24_O_31_Cl_10_ (**a**), the possible charge transfer of photogenerated electron-hole pairs (**b**) and the transient photocurrent response of samples B-RT, B-450 and B-600.

To investigate the photocatalytic process in detail, the relative conduction band (CB) and valence band (CB) potentials of the semiconductors should be determined. The Mott-Schottky plots of B-RT (BiOCl) and B-600 (Bi_24_O_31_Cl_10_) are shown in Figure S8. It is found that the flat-band potential (*V*_fb_) of BiOCl and Bi_24_O_31_Cl_10_ are determined to be 0.46 and −0.33 V *versus* Ag/AgCl (equivalent to 0.68 and −0.11 V *versus* NHE) through extrapolating the linear parts of the Mott-Schottky plots to potential axis, respectively. It is generally known that the conduction band potentials (*E*_CB_) of n-type semiconductors are very close to (0.1 ~ 0.2 eV more negative) the flat-band potentials^60^. Thus, we could deduce that the CB position of Bi_24_O_31_Cl_10_ (−0.21 eV) is more negative than that of BiOCl (0.58 eV). The schematic band diagrams of pure BiOCl and Bi_24_O_31_Cl_10_ are illustrated in Fig. 8a.

The charge transfer in the Bi_24_O_31_Cl_10_/BiOCl heterojunction is depicted in Fig. 8b. The electrons are excited from VB of Bi_24_O_31_Cl_10_ to the CB potential position (−0.21 eV) under visible light irradiation, but the electrons in the VB of BiOCl could not be excited because of its wide band gap. Partial photogenerated electrons transfer to the CB of BiOCl and the other part would be trapped by O_2_ to produce O_2_^−^ radicals because of the less redox potential (−0.16 eV)^61^ of O_2_/^•^O_2_^−^. The photogenerated holes in the VB of Bi_24_O_31_Cl_10_ react with benzyl alcohol and convert them to benzaldehyde. The generation of O_2_^−^ radicals greatly inhibits the recombination of photogenerated charge carriers, which is favorable for the photocatalytic performance.

To confirm the efficient separation of photogenerated charge carriers, photocurrent transient response measurements of sample B-RT, B-450 and B-600 are performed (Fig. 8c). As shown in Fig. 8c, all samples are prompt in producing photocurrent with a reproducible response to on/off cycle under visible light irradiation, suggesting that absorption of light could produce the photo-induced charge carriers and the charge carriers could transfer effectively. In comparison with B-RT and B-600, the sample B-450 displays the strongest peak intensity, implying more excellent photocatalytic activity of the Bi_24_O_31_Cl_10_/BiOCl heterojunction than the sole semiconductor counterparts.

## Conclusions

A Bi_24_O_31_Cl_10_/BiOCl heterojunction has been successfully constructed through a simple thermal annealing route. Various characterization techniques confirm the construction of the Bi_24_O_31_Cl_10_/BiOCl heterojunction during the annealing process. The obtained Bi_24_O_31_Cl_10_/BiOCl photocatalyst displays excellent photocatalytic efficiency and selectivity toward the conversion of benzyl alcohol to benzaldehyde under visible light irradiation, which could reach 40.3% and >99%, respectively. The photogenerated holes play an important role in the photocatalytic oxidation of benzyl alcohol and superoxide radicals are not involved in the photocatalytic process. The *in-situ* generation of heterojunction photocatalysts may provide superior interfacial contact, which is advantageous for enhancing the photocatalytic performance.

## Methods

### Bi_24_O_31_Cl_10_/BiOCl heterojunction synthesis

All chemical solvents and reagents were analytical grade and were used without further purification. In a typical procedure, 0.776 g Bi(NO_3_)_3_·5H_2_O was dissolved in 76 mL of glycerol, denoted as solution A. Then, 0.12 g KCl was dissolved in 4 mL of deionized water (solution B), which was subsequently poured into solution A. After stirring for 15 min, the mixture was transferred into a 100 mL Teflon-lined stainless steel autoclave, heated to 110 °C and kept at this temperature for 8 h. The resulting precipitate was collected by centrifugation, then washed with ethanol and deionized water for several times, and dried at 80 °C in vacuum to obtain the pure BiOCl powder (denoted as B-RT).

The thermal annealing step was performed in an air-atmosphere programmable tube furnace in the temperature range of 400 ~ 600 °C with an interval of 50 °C. The final products were denoted as B-400 ~B-600, respectively.

## Characterization

Detailed crystallographic information of the synthesized samples was obtained on an X-ray diffractometer (Empypean Panalytical) with Cu K*a* radiation (*λ* = 0.15406 nm). The thermogravinetric analysis (TG) and differential thermal analysis (DTA) were carried out on a thermal analyzer (NETZSCH STA 449F3) where the sample was heated from 30 to 950 °C with a raising ramp rate of 10 °C/min under nitrogen atmosphere. The detailed morphology, structure and heterojunction feature of the samples were recorded by transmission electron microscopy (TEM) and high resolution TEM (HRTEM) on a JEM-2010 apparatus with an acceleration voltage of 200 kV. The surface state and chemical composition of the samples were analyzed by X-ray photoelectron spectroscopy (XPS), which was carried out on a Thermo Escalab 250Xi with a monochromatic Al Ka (*hv* = 1486.6 eV). Raman spectra were recorded on the Horiba Jobin Yvon LabRAM HR800 instrument with the laser excitation of 532 nm. Fouier transform infrared spectroscopy (FT-IR) was performed using a Bruker Tensor 27 spectrophotometer using KBr powder-pressed pellets. The UV-vis absorption spectra were measured using a UV-vis spectrophotometer (Lambda 750s) in the range of 200 ~ 800 nm. The specific surface area (*S*_BET_) of the samples was obtained from N_2_ adsorption-desorption isotherms at 77 K (ASAP 2020). Prior to the sorption experiment, the materials were dehydrated by evacuation under specific conditions (200°C, 10 h).

The photocurrent transient response measurement was carried out based on a lock-in amplifier. The measurement system is constructed by a sample chamber, a lock-in amplifier (SR 830, Stanford Research Systems, Inc.) with a light chopper (SR540, Stanford Research Systems, Inc.) and a source of monochromatic light which is provided by a 500 W xenon lamp (CHF-XM 500, Trusttech) and a monochromator (Omni-*λ*300, Zolix). The monochromator and the lock-in amplifier were equipped with a computer. The analyzed product is assembled as a sandwich-like structure of ITO-product-ITO, which ITO means an indium tin oxide electrode. All the measurements were performed in air atmosphere and at room temperature.

Electron spin resonance (ESR) spectra were obtained on a Brüker ER200-SRC apparatus. A frequency of about 9.06 GHz was used for a dual-purpose cavity operation. The magnetic field of 0.2 mT was modulated at 100 kHz. A microwave power of about 1 mW was employed. Other parameters for the apparatus were set at: sweep width of 250 mT, center field of 250 mT, sweep time of 2.0 min, and accumulated 5times. All measurements were performed at room temperature in air without vacuum-pumping. ESR spectra for hydroxyl radicals and superoxide radicals were conducted in methylbenzene solution (2.0 mL) and methylbenzene solution containing methyl alcohol (2 mL, the volume ratio of methyl alcohol being 20%), respectively. The experiments were processed in dark and under visible light irradiation with adding 4 mg sample and 0.05 M DMPO.

All calculations were performed with density functional theory (DFT), using the CASTEP program package. The kinetic energy cutoff is 420 eV, using the generalized gradient approximation (GGA) with the Perdew-Burke-Ernzerhof (PBE) to treat the models. Geometry optimization is carried out until the residual forces were smaller than 0.01 eV Å^−1^, and the convergence threshold for self-consistent iteration was set at 5 × 10^−7^ eV.

### Photocatalytic activity Test

Selective Oxidation of benzyl alcohol has been widely studied as a model reaction to estimate the photocatalytic performance of catalysts. The photocatalytic activity experiments were carried out in a photochemical reactor fitted with a 500 W xenon lamp and a visible-light optical filter (λ > 420 nm). 10 mL methylbenzene solution involving alcohol (1 mM) mixed with 0.05 g sample was magnetically stirred at 25 °C in water bath. Anaerobic and aerobic reactions were performed by bubbling with pure N_2_ and O_2_, respectively, for at least 1 hour before visible-light irradiation. After illuminating 10 hours, the suspension was centrifuged to remove the powder and measured the concentration of the alcohol and product by GC-FID (Shimadzu GC-2014C).

## Additional Information

**How to cite this article**: Liu, X. *et al*. Constructing Bi_24_O_31_Cl_10_/BiOCl heterojunction via a simple thermal annealing route for achieving enhanced photocatalytic activity and selectivity. *Sci. Rep.* **6**, 28689; doi: 10.1038/srep28689 (2016).

## Supplementary Material

Supplementary Information
