# Supplementary material for: Constructing Bi24O31Cl10/BiOCl heterojunction via a simple thermal annealing route for achieving enhanced photocatalytic activity and selectivity
Source: Sci Rep. 2016 Jun 24;6:28689. doi: 10.1038/srep28689 (PMC4919631; doi:10.1038/srep28689)
Supplement: Supplementary Information [file srep28689-s1.doc]

Electronic Supporting Information (ESI) for

Constructing Bi_24_O_31_Cl_10_/BiOCl heterojunction via a simple thermal annealing route for achieving enhanced photocatalytic activity and selectivity

Xiaoyan Liu, Yiguo Su, Qihang Zhao, Chunfang Du*, Zhiliang Liu*

College of Chemistry and Chemical Engineering, Inner Mongolia University, Hohhot, Inner Mongolia 010021, P. R. China

*Corresponding author. Tel.: +86-471-4995414; Fax: +86-471-4995414.

E-mail address: cedchf@imu.edu.cn (C. Du), cezlliu@imu.edu.cn (Z. Liu).

**ESI-1**


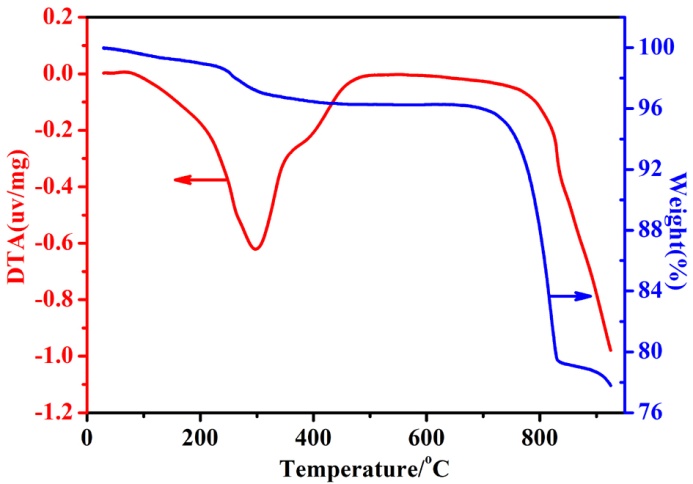


Figure S1 DTA-TG curves of sample B-RT

**ESI-2**


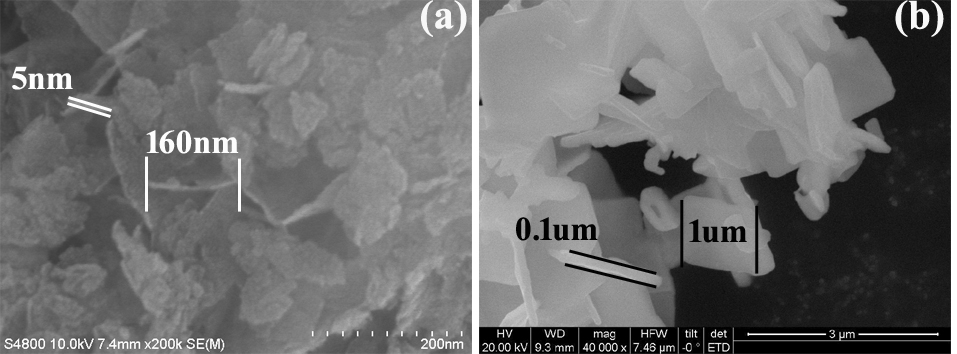


Figure S2 SEM images of sample B-RT(a) and B-600(b)

**ESI-3**


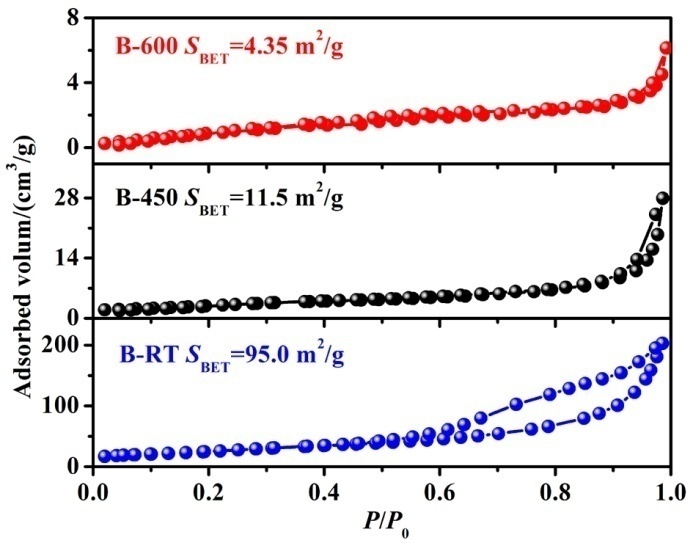


Figure S3 N_2_ adsorption-desorption isotherms of B-RT, B-450 and B-600.

**ESI-4**

Table S1 Chemical compositions of various samples from the XPS results

| Samples | Bi/at% | Cl/at% | O/at% | Bi/Cl molar ratio |
| --- | --- | --- | --- | --- |
| B-RT | 19.604 | 20.071 | 31.607 | 0.977 |
| B-400 | 23.475 | 23.177 | 35.744 | 1.013 |
| B-450 | 23.164 | 19.765 | 34.710 | 1.172 |
| B-500 | 22.290 | 16.266 | 39.530 | 1.370 |
| B-550 | 21.202 | 11.496 | 41.845 | 1.844 |
| B-600 | 21.852 | 9.518 | 38.161 | 2.296 |

**ESI-5**


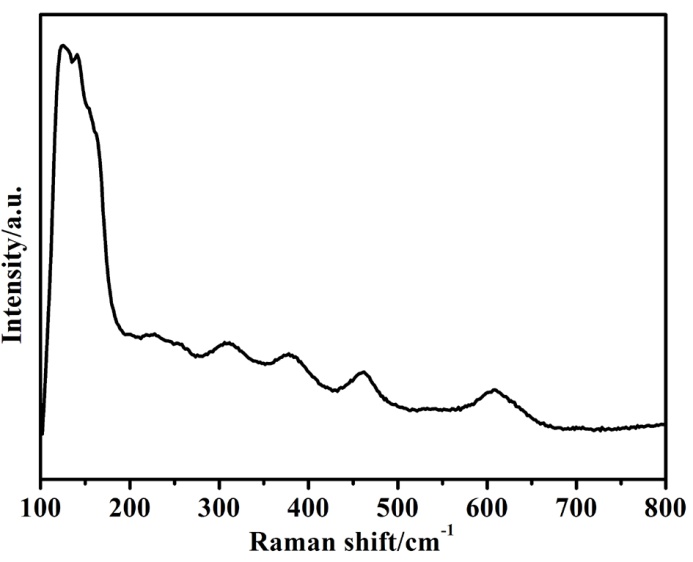


Figure S4 Raman spectra of Bi_24_O_31_Cl_10_ synthesized according to the reference

**ESI-6**


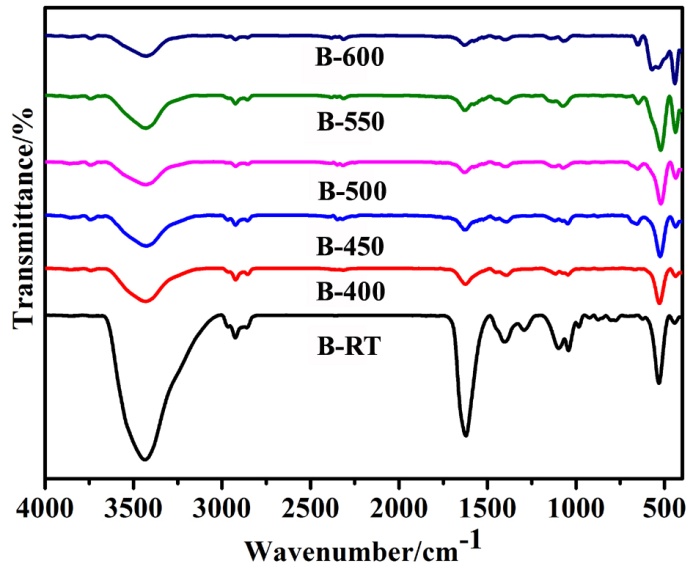


Figure S5 FT-IR spectra of various samples

**ESI-7**


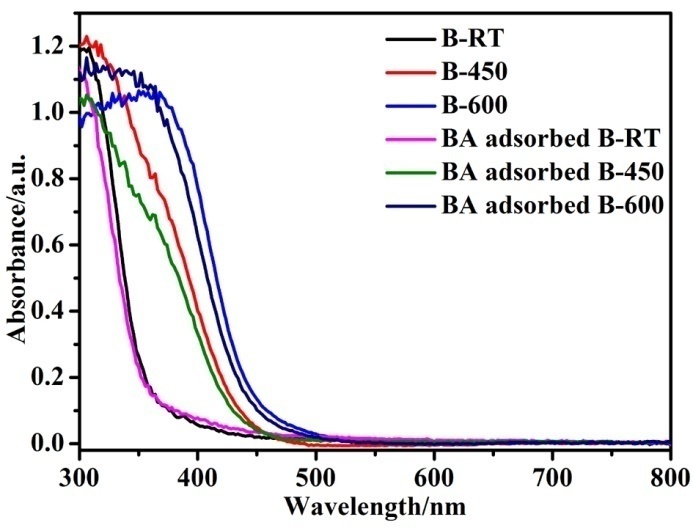


Figure S6 UV-vis diffuse reflectance spectra of B-RT, B-450, B-600 and benzyl alcohol (BA)-adsorbed counterparts

The benzyl alcohol adsorption experiments were conducted according to the reference^1^. Various amples were first suspended in benzyl alcohol solution with acetonitrile as solvent under magnetic stirring condition in darkness for 12 h, and then separated by microfiltration and dried at 60 ^◦^C for 24 h. Followingly, the UV-vis diffuse reflectance spectra of various samples were measured.

References

1.Li, C. J., Xu, G. R., Zhang, B. & Gong, J. R. High selectivity in visible-light-driven partial photocatalytic oxidation of benzyl alcohol into benzaldehyde over single-crystalline rutile TiO_2_ nanorods. *Appl. Catal. B: Environ.* **115-116**, 201-208 (2012).

**ESI-8**

Table S2 The comparsion of selective photocatalytic oxidation of benzyl alcohol to benzaldehyde over the resultant samples and other samples reported in references

| Samples | Conversion/% | Selectivity/% | Light sources | Refs |
| --- | --- | --- | --- | --- |
| B-450 | 40.3 | >99 | 500 W Xe lamp with a 420 nm cut-off filter | This work |
| P25 | 59.5 | 70~85 |  | 7 |
| g-C_3_N_4_ | 22.1 | >99 |  | 7 |
| Bi_3_O_4_Br | 36.0 | >99 |  | 7 |
| BiOBr | 21.6 | >99 |  |  |
| Bi_12_O_17_Cl_2_ | 44.0 | >99 |  | 7 |
| Na*_x_*TaO*_y_*·*n*H_2_O | 63.3 | >99 | 300 W mercury lamp with a 420nm cut off filter | 1 |
| TiO_2_ | >99 | >99 | LED lamp | 19 |

**ESI-9**


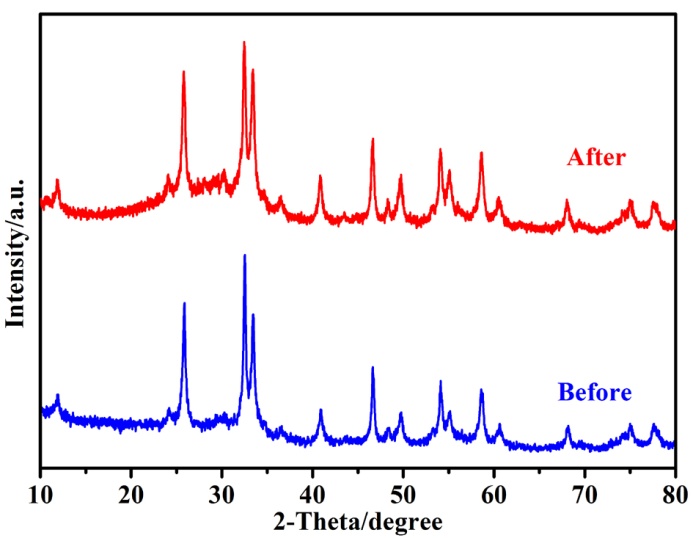


Figure S7 XRD patterns of sample B-450 before and after photocatalytic reaction

**ESI-10**


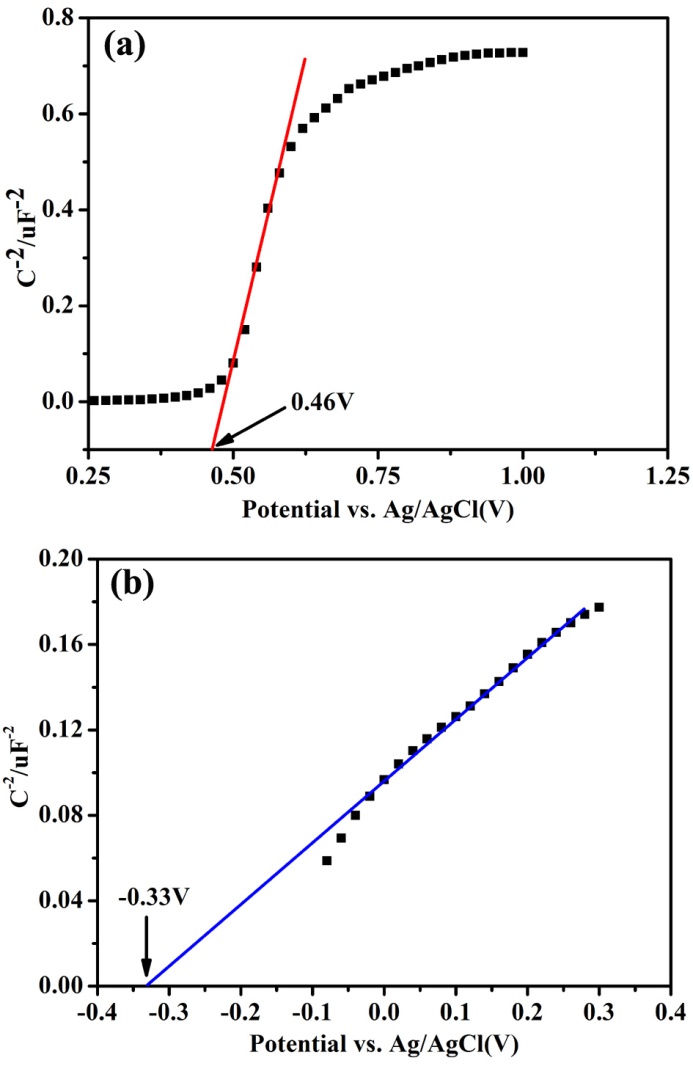


Figure S8 The Mott-Schottky plots of B-RT (a, BiOCl) and B-600 (b, Bi_24_O_31_Cl_10_)

The flat-band potentials of the semiconductors were determined from the Mott–Schottky plots on a BIOLOGIC VSP300 electrochemical workstation. The electrolyte was 0.2 M Na_2_SO_4_ solution. A Xe-arc lamp with a 420 nm cutoff filter was employed as a visible light photo source, and the light intensity was set to be 100 mW/cm^2^. The reactor was the three-electrode electrochemical cell including a working electrode, a platinum mesh counter electrode and a Ag/AgCl (3 M KCl) as reference electrode.
